# Retrospective Analysis of COVID-19 Conversion Rate Among Anesthesiologists in Acute Care Centers

**DOI:** 10.7759/cureus.17131

**Published:** 2021-08-12

**Authors:** Michael Yakobi, Harish Nandigam, James Fallon

**Affiliations:** 1 Emergency Department, Thomas Jefferson University Hospital, Philadelphia, USA; 2 Anesthesiology, Saint Michael's Medical Center, Newark, USA; 3 Clinical Research, Saint Michael's Medical Center, Newark, USA

**Keywords:** academic anesthesiology, anesthesia education, covid 19, acute care community hospitals, infection rates

## Abstract

Brief description of the primary research objective

Among healthcare workers, anesthesiologists are regarded as frequently exposed frontline providers in the fight against COVID-19 due to their proximity to patient airways and involvement in aerosolized procedures. As such, the risk of contracting the COVID-19 virus as an occupational hazard is presumed to be higher. To date, in most published studies, all healthcare workers were grouped together, independent of specialty or profession. At the time that this survey was distributed, we did not find any peer-reviewed articles that differentiated COVID-19 infection rates among frontline, such as anesthesiologists vs. non-frontline healthcare workers. This retrospective survey’s primary research objective was to report the rate of COVID-19 infection among anesthesiologists compared to the general population of healthcare workers.

Methodology

A survey was sent among anesthesiology attendings and residents in Northern New Jersey and Brooklyn, New York hospitals on duty during the peak pandemic from March 2020 to May 2020. Questions in the survey focused on infection rates and adherence to standards of infection precaution and personal protective equipment (PPE) utilization.

Main Findings

This retrospective study highlights the rate of infection among anesthesiologists as a particularly vulnerable subgroup of frontline residents and physicians, as they are called to duty when emergent airway management is required. In our study, the reported rate of contracting COVID-19 among anesthesiologists was 16.7%. This statistic is higher than the infection rates published by studies by New York State and the CDC.

Conclusion

The survey sent to anesthesiologists is useful to understand the impact of COVID-19 on this subgroup of frontline providers and the importance of adhering to standards of infection protocol and the role of PPE.

## Introduction

During the COVID-19 pandemic, healthcare workers are considered a high-risk group for contracting the virus. For those on the frontline, the risk is even higher. A subgroup of frontline healthcare workers, anesthesiologists, are particularly vulnerable. As experts in airway management, they are frequently asked to manage emergent airways and secure the respiratory tract, exposing them to droplet and airborne pathogens.

There are many articles and reports on the COVID-19 infection rate among healthcare workers. The reported rates vary from 1% in studies conducted in Wuhan, China [1], to a Dutch study that found 6.4% of symptomatic employees in the healthcare field tested positive for the COVID-19 infection, which represented 0.9% of all healthcare workers [2,3]. In April 2020, CDC officials provided data suggesting that healthcare workers accounted for about 11% of COVID-19 infections worldwide [4]. The office of New York State Governor Andrew Cuomo reported that 12% of healthcare workers in Downstate New York tested positive for the COVID-19 virus [5], while in Ohio, infection rates among healthcare workers were 18% during the same period [6]. Another publication of meta-analysis review of over 2000 articles of the National Library of Medicine MEDLINE database revealed that in total, healthcare workers accounted for 2.5% of total infections. Although findings indicated that the majority of cases were mild, 14.5% were considered severe or critical, and healthcare worker-specific mortality was 1.3% [7].

## Materials and methods

We conducted a retrospective study among anesthesiologists to better assess the COVID-19 conversion rate among anesthesiologists. Based on the replies the research team received in response to emailed requests for participants, the hospitals involved in this study are in Northern New Jersey and Brooklyn, New York hospitals. Hospitals that participated in the study are located in the cities of Newark and Paterson in New Jersey and Brooklyn, New York. All are acute care community teaching hospitals with 120, 240, and 370-bed capacity, respectively. During the peak pandemic period from mid-March 2020 to mid-May 2020, as few as 575 COVID-19 positive patients were hospitalized in the 120-bed hospital, and as many as 1,000 COVID-19 positive patients were hospitalized in the 370-bed hospital.

An original survey, please see Appendix A, was created by the authors. The survey was then distributed via email among anesthesiology assistants, attendings, nurse anesthetists, and residents that were on duty during the above-mentioned peak pandemic period. However, only attending anesthesiologists and residents responded. No incentive, financial or otherwise, was provided to those who responded. The ten-question, multiple choice survey focused on the presence of symptoms, hours worked, the location where duty hours took place (i.e., OR, ICU, ward, ER), and on adherence to standards of infection precaution and personal protective equipment (PPE) utilization. The results of the survey were then analyzed by the director of clinical research at Saint Michael’s Medical Center, James Fallon. The mean and CI for each response were analyzed and reported below.

No patient information of identifiers or any kind was provided in the results analyzed. As such, the investigators are confident that there was no violation of the patient's confidentiality, as this was a retrospective survey that did not include the name, date of birth, or past medical history of the responder.

## Results

Surveys were sent to 87 anesthesia attendings and residents, of which 42 responded. These responses were entered into a Quality Improvement (QI) database. All 42 providers (100%) wore PPE and adhered to standards of infection control during the management of acute airway as set forth by various anesthesia associations, including the American Society of Anesthesiologists (ASA), Anesthesia Patient Safety Foundation (APSF), American Academy of Anesthesiologist Assistants (AAAA), and American Association of Nurse Anesthetists (AANA).

All institutions in the study offered providers to be tested for COVID-19 with polymerase chain reaction (PCR) or antibody (Ab) test. Out of 42 responders, seven, i.e., 16.7% tested positive by PCR or AB. Out of seven anesthesiologists, six experienced either fever, cough, or GI symptoms. Forty-one providers worked full-time hours anywhere between 40 and 50 hours per week, except one provider who worked 20-30 hours per week. Out of seven anesthesiologists that tested positive, only one worked 20-30 hours per week. The results are highlighted in Table 1.

**Table 1:** Results of administered survey.

Number of providers' responses (n = 42)	Tested for COVID-19 (%)	Did not test for COVID-19 (%)	Tested positive for COVID-19 (%)	Symptomatic COVID-19 positive (%)	Hours worked per week among symptomatic and asymptomatic providers	Adhered to standards of infection precaution (%)
42	64 [95% CI: (53.5-74.5)]	36 [95% CI: (25.5-46.5)]	16.7 [95% CI (8.54-24.86)]	14.3 [95% CI: (6.64-21.96)]	20-50	100

Intubations, airway management, pre, and post-operative care were performed on the floors, ICUs, and EDs. The percentage of intubations among the above institutions is unknown, but one study revealed that among 5700 patients hospitalized with COVID-19 in New York, 1151 (20%) required mechanical ventilation [8].

## Discussion

This study has few limitations. One is that it is hard to ascertain where physicians contracted the COVID-19 virus, as it became ubiquitous and present both in a community and in the hospital setting. To the best of our knowledge, no article was able to accurately differentiate where the exposure and virus contraction had occurred.

Another limitation was the difficulty in determining with certainty how many intubations each provider performed to see if any correlation between conversion to infection and the number of intubations performed exist. For the same reasons, it was not possible to definitively determine if any correlation exists between intubation site (ER, OR, ICU, ward) and infection with COVID-19. Lastly, given patient confidentiality, it is not possible to determine if those who were infected had predisposing risk factors, making them vulnerable to infection.

When a pathogen transmitted via respiratory droplets, such as the COVID-19 virus, is suspected, the care anesthesiologists provide imposes a significant hazard to their own health [9]. Healthcare workers in general, and anesthesiologists in particular, are at high risk given their physical proximity to airways and involvement in aerosolizing procedures. Careful planning and strict adherence to clinical practice guidelines published for anesthesiology are essential in minimizing the risk of infection [10]. This includes those set forth by the American Society of Anesthesiologists (ASA), Anesthesia Patient Safety Foundation (APSF), American Academy of Anesthesiologist Assistants (AAAA), and American Association of Nurse Anesthetists (AANA) [11,12,13].

## Conclusions

Anesthesiologists are called to treat patients as experts in emergent airway management in ICUs, EDs, the wards, as well as in perioperative and operative settings. Given the physical proximity to potential airborne viruses and their significant involvement in aerosolized procedures that further the spread of these viruses, the risk of contracting viruses like COVID-19 is presumed to be higher among anesthesiologists compared to other fields of medicine. To our knowledge, this study represents the first study that focuses on the COVID-19 infection rate among anesthesiologists in acute care hospitals during the peak COVID-19 pandemic of March 2020 to May 2020. Therefore, in this study, we aimed to examine if the rate of contracting COVID-19 was different when compared to other frontline healthcare workers. In our study, the reported rate of contracting COVID-19 was 16.7%. This statistic is higher than the infection rates for the general population of healthcare workers published by the above-mentioned studies published by the New York State and the CDC. This finding supports our initial hypothesis.

Additionally, this study’s secondary finding of 100% self-reported adherence to standards of infection precautions is a testament to the work of anesthesia societies, which shared data and evidence to encourage the proper use of PPE in clinical settings. The educational efforts of these organizations provided significant protection and likely resulted in a lower conversion rate than what would have been observed without their efforts and sharing of evidence. Given that anesthesiologists are among high-risk providers to contract the virus, emphasizing the importance of adhering to the standards of infection precautions and wearing proper PPE, it is possible to mitigate the rate of infection among frontline healthcare workers.

## Appendices

1.     Institution. Please type your answer:

2.     What is your profession?

a.     Anesthesiologist assistant

b.     Attending anesthesiologist

c.     Nurse anesthetist

d.     Resident anesthesiologist

3.    Did you perform anesthesiologist clinical duties, including but not limited to intubations, during the peak of the COVID-19 pandemic (March 2020May 2020)?

a.     Yes

b.     No

4.     How many hours did you work per week during this period, on average?

a.     0-10

b.     10-20

c.     20-30

d.     30-40

e.     40-50

f.      50-60

g.     60-70

h.     70-80

i.       80+

5.     Which PPE was provided by your institution? Please select all that apply.

a.     N95

b.     Face shield

c.     Goggles

d.     Disposable coveralls

e.     Other, please specify:

6.     Did PPE provided by your institution meet the standards as set by the American Society of Anesthesiologists (ASA), Anesthesia Patient Safety Foundation (APSF), American Academy of Anesthesiologist Assistants (AAAA), and American Association of Nurse Anesthetists (AANA)?

a.     Yes

b.     No

c.     Not sure

7.     Did you have any symptoms? Please select all that apply.

a.     No, I did not experience any symptoms

b.     Fever

c.     Cough

d.     Dyspnea

e.     Olfactory loss

f.      Other, please specify:

8.     Were you ever tested for COVID-19?

a.     No, I was not tested

b.     Yes, by antigen/PCR nasopharyngeal swab

c.      Yes, by an antibody blood test

d.     Yes, by another method

9.     If tested, what was the result? Please type your answer:

10.   In performing your clinical duties, where did you intubate patients? Please select all that apply.

a.     ED

b.     ICU

c.     Floor

d.     Operating room

e.     Other, please specify
